# A stratification strategy to predict secondary infection in critical illness-induced immune dysfunction: the REALIST score

**DOI:** 10.1186/s13613-022-01051-3

**Published:** 2022-08-17

**Authors:** Jan-Alexis Tremblay, Florian Peron, Louis Kreitmann, Julien Textoris, Karen Brengel-Pesce, Anne-Claire Lukaszewicz, Laurence Quemeneur, Christophe Vedrine, Lionel K. Tan, Fabienne Venet, Thomas Rimmele, Guillaume Monneret, the REALISM study group, Sophie Arnal, Caroline Augris-Mathieu, Frédérique Bayle, Liana Caruso, Charles-Eric Ber, Asma Ben-amor, Anne-Sophie Bellocq, Farida Benatir, Anne Bertin-Maghit, Marc Bertin-Maghit, André Boibieux, Yves Bouffard, Jean-Christophe Cejka, Valérie Cerro, Jullien Crozon-Clauzel, Julien Davidson, Sophie Debord-Peguet, Benjamin Delwarde, Robert Deleat-Besson, Claire Delsuc, Bertrand Devigne, Laure Fayolle-Pivot, Alexandre Faure, Bernard Floccard, Julie Gatel, Charline Genin, Thibaut Girardot, Arnaud Gregoire, Baptiste Hengy, Laetitia Huriaux, Catherine Jadaud, Alain Lepape, Véronique Leray, Anne-Claire Lukaszewicz, Guillaume Marcotte, Olivier Martin, Marie Matray, Delphine Maucort-Boulch, Pascal Meuret, Céline Monard, Florent Moriceau, Guillaume Monneret, Nathalie Panel, Najia Rahali, Thomas Rimmele, Cyrille Truc, Thomas Uberti, Hélène Vallin, Fabienne Venet, Sylvie Tissot, Abbès Zadam, Sophie Blein, Karen Brengel-Pesce, Elisabeth Cerrato, Valerie Cheynet, Emmanuelle Gallet-Gorius, Audrey Guichard, Camille Jourdan, Natacha Koenig, François Mallet, Boris Meunier, Virginie Moucadel, Marine Mommert, Guy Oriol, Alexandre Pachot, Estelle Peronnet, Claire Schrevel, Olivier Tabone, Julien Textoris, Javier Yugueros Marcos, Jeremie Becker, Frederic Bequet, Yacine Bounab, Florian Brajon, Bertrand Canard, Muriel Collus, Nathalie Garcon, Irene Gorse, Cyril Guyard, Fabien Lavocat, Philippe Leissner, Karen Louis, Maxime Mistretta, Jeanne Moriniere, Yoann Mouscaz, Laura Noailles, Magali Perret, Frederic Reynier, Cindy Riffaud, Mary-Luz Rol, Nicolas Sapay, Trang Tran, Christophe Vedrine, Christophe Carre, Pierre Cortez, Aymeric Monfort, Karine Florin, Laurent Fraisse, Isabelle Fugier, Sandrine Payrard, Annick Peleraux, Laurence Quemeneur, Andrew Griffiths, Stephanie Toetsch, Teri Ashton, Peter J Gough, Scott B Berger, David Gardiner, Iain Gillespie, Aidan Macnamara, Aparna Raychaudhuri, Rob Smylie, Lionel Tan, Craig Tipple

**Affiliations:** 1 EA 7426 “Pathophysiology of Injury-Induced Immunosuppression” (Université Claude Bernard Lyon 1 - Hospices Civils de Lyon - bioMérieux), Joint Research Unit HCL-bioMérieux, Herriot Hospital, 5 place d’Arsonval, 69003 Lyon, France; 2 Critical Care Service, Hôpital Maisonneuve-Rosemont, 5415 Boulevard de l’Assomption, Montréal, H1T2M4 Canada; 3 Anesthesia and Critical Care Medicine Department, Hospices Civils de Lyon, Edouard Herriot Hospital, 69437 Lyon, France; 4 Sanofi Pasteur, Sanofi 1541 avenue Marcel Mérieux, 69280 Marcy l’Etoile, France; 5 BIOASTER, 40 Avenue Tony Garnier, 69007 Lyon, France; 6 GSK, 980 Great West Road, Brentford, Middlesex TW8 9GS UK; 7 Immunology Laboratory, Hospices Civils de Lyon, Edouard Herriot Hospital, 69437 Lyon, France; 8 Centre International de Recherche en Infectiologie (CIRI), Inserm U1111, CNRS, UMR5308, Ecole Normale Supérieure de Lyon, Team ‘NLRP3 Inflammation and Immune Response to Sepsis’, Université Claude Bernard-Lyon 1, Lyon, France

**Keywords:** Sepsis, HLA-DR, IL-10, Neutrophil, Immunosuppression, Critical care, Secondary infection

## Abstract

**Background:**

Although multiple individual immune parameters have been demonstrated to predict the occurrence of secondary infection after critical illness, significant questions remain with regards to the selection, timing and clinical utility of such immune monitoring tests.

**Research question:**

As a sub-study of the *REALISM* study, the *REALIST* score was developed as a pragmatic approach to help clinicians better identify and stratify patients at high risk for secondary infection, using a simple set of relatively available and technically robust biomarkers.

**Study design and methods:**

This is a sub-study of a single-centre prospective cohort study of immune profiling in critically ill adults admitted after severe trauma, major surgery or sepsis/septic shock. For the REALIST score, five immune parameters were pre-emptively selected based on their clinical applicability and technical robustness. Predictive power of different parameters and combinations of parameters was assessed. The main outcome of interest was the occurrence of secondary infection within 30 days.

**Results:**

After excluding statistically redundant and poorly predictive parameters, three parameters remained in the *REALIST* score: mHLA-DR, percentage of immature (CD10^−^ CD16^−^) neutrophils and serum IL-10 level. In the cohort of interest (*n* = 189), incidence of secondary infection at day 30 increased from 8% for patients with *REALIST* score of 0 to 46% in patients with a score of 3 abnormal parameters, measured ad D5–7. When adjusted for a priori identified clinical risk factors for secondary infection (SOFA score and invasive mechanical ventilation at D5–7), a higher *REALIST* score was independently associated with increased risk of secondary infection (42 events (22.2%), adjusted HR 3.22 (1.09–9.50), *p* = 0.034) and mortality (10 events (5.3%), *p* = 0.001).

**Interpretation:**

We derived and presented the *REALIST* score, a simple and pragmatic stratification strategy which provides clinicians with a clear assessment of the immune status of their patients. This new tool could help optimize care of these individuals and could contribute in designing future trials of immune stimulation strategies.

**Graphical Abstract:**

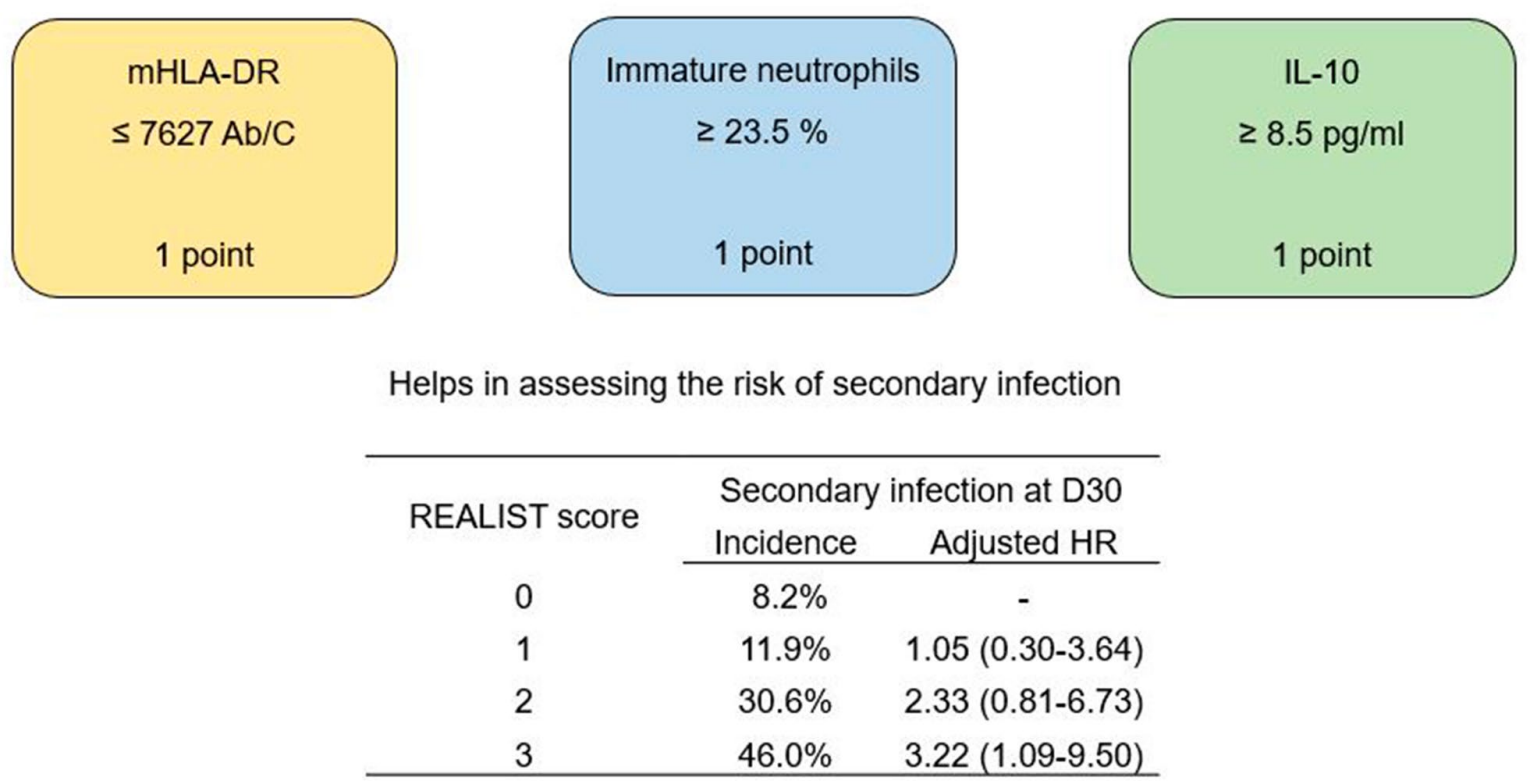

**Supplementary Information:**

The online version contains supplementary material available at 10.1186/s13613-022-01051-3.

## Introduction

For patients with critical illness, occurrence of secondary infection is a major and frequent complication, affecting between 15 and 40% of patients after an Intensive Care Unit (ICU) admission [1–6]. Such infections are associated with increased morbidity and mortality and represent a high burden of care with longer ICU length of stay and overall greater healthcare costs [2, 7]. In addition, they contribute to higher rates of microbial resistance through extensive use of antibiotic and antifungal agents, a pressing and worldwide issue [8–10] which has recently been further highlighted amidst the COVID-19 pandemic [11].

Among factors leading to acquisition of secondary infection in the ICU, the contribution of critical illness-induced immune dysfunction is now well recognized. Although this phenomenon, which affects both innate and adaptive immune responses, has been mainly described in sepsis [12, 13], similar immune alterations have been described in various aetiologies of critical illness [14–16], suggesting a somewhat common immune pathway. The REALISM study [17] (REAnimation Low Immune Status Marker) was performed to describe deep immune profiling of injury-induced immune response in a variety of critical illnesses, and among other findings has further reinforced the concept of a common global immune response to various types of severe injury.

Although multiple immune parameters have been shown to have some degree of predictive power for occurrence of secondary infection, significant heterogeneity exists regarding which test to use, with which cutoff values, at which timepoint and in which population [14, 18]. As such, there is a need for clinically relevant stratification tools to assess the occult immune status of critically ill patients to better tailor care of such fragile individuals. Of note, in REALISM, the occurrence of secondary infection was somehow late (median day 9 [6–15] after ICU admission) and predominantly occurred in patients who were still in the ICU.

As a sub-study of REALISM, the REALIST score was thus developed as a pragmatic approach to help clinicians better identify and stratify patients at high risk for secondary infection after the initial phase of resuscitation, using a simple set of relatively commonly available and technically robust biomarkers. The main objective of this study is to explore the predictive power of the REALIST score regarding subsequent secondary infection.

## Methods

This is a sub study of REALISM [17], for which a detailed protocol has been previously published [19]. In summary, REALISM is a prospective, observational cohort study of critically ill patients admitted with sepsis, severe trauma or planned surgery, which was performed from 2015 to 2018 at the Edouard Herriot Hospital (Hospices Civils de Lyon, France). The study protocol was approved by institutional review board (Comité de Protection des Personnes Sud-Est II) under number 2015-42-2.

Inclusion criteria for REALISM were: adult patients admitted to the ICU with a clinical diagnosis of sepsis as defined by 2016 SEPSIS-3 consensus guidelines [20]; or severe trauma with injury severity score (ISS) > 15; or surgical patients undergoing major surgeries, such as eso-gastrectomy, bladder resection with Brickers’ reconstruction, cephalic pancreaticoduodenectomy and abdominal aortic aneurysm surgery by laparotomy. Exclusion criteria were any of the following: presence of a pre-existent condition or under treatment that could influence patients’ immune status, pregnancy, institutionalized patients and inability to obtain informed consent. Written informed consent was obtained from every patient or their representative upon inclusion in this protocol. In the event that only the informed consent of a third party has been sought at the time of inclusion, the patients were informed as soon as possible of their participation in this study and asked to give their own consent to continue the study.

### Sampling and clinical data collection

Regarding the present sub-study, samples were collected three times during the first week after enrollment: at day 1 or 2 (D1–2), D3–4 and D5–7, with the latter pre-emptively selected as the timepoint of interest. Peripheral whole blood was collected in one ethylenediaminetetraacetic acid **(**EDTA) tube at each timepoint for each patient. Tubes were immediately transferred to the lab and processed within 3 h after blood sampling for flow cytometry immune phenotyping and plasma cytokine level measurements.

The main cohort consisted of all patients initially enrolled in the REALISM study. As most secondary infections in the ICU occur more than 1 week after admission (median 9 [6–15] days in the REALISM study) and in an effort to select patients with persistently high risk of events, a predefined cohort of interest was formed, consisting of all patients who were still alive and in the ICU at D5–7. Patients who developed a secondary infection prior to their sampling day were excluded.

Patients’ demographics, comorbidities, diagnosis, severity and clinical outcomes were prospectively collected and longitudinal follow-up was performed for 90 days. The following data were recorded: demographic information (age, gender, body mass index (BMI)), disease severity measured by the Simplified Acute Physiological Score (SAPS) II at ICU admission [21] and the Sequential Organ Failure Assessment (SOFA) score [22] measured at D1, D3–4 and D5–7. ISS was collected for trauma patients [23]. Hospital and ICU lengths of stay and survival were measured until day 90 after admission. Follow-up location after ICU discharge was recorded. During hospital stay, patients were screened daily for exposure to invasive mechanical ventilation and for secondary infection occurrence. The main outcome of interest was the occurrence of secondary infection within day 30 after ICU admission and prespecified secondary outcomes were mortality at day 30, days free from ICU at 30 days and days free from hospital at 30 days.

### Definition of secondary infections

Information related to infections were collected by research nurses and reviewed and validated by a dedicated adjudication committee composed of 3 clinicians not involved in patients’ recruitment or care and scrutinizing data simultaneously. Confirmation of secondary infection occurrence by this committee was based on guidelines defined by the European Center for Disease Prevention and Control [24] and Infectious Diseases Society of America [25]. “Definite” and “likely” infections were included and only the first secondary infection episode was considered in the analyses. Adjudication committee was blinded for results of immune parameters. Patients who died without being identified as having a “definite” or “likely” secondary infection were not censored from analysis.

### Score derivation

Five biological parameters were initially selected for analysis, based on their established association with critical illness immune perturbations, availability and cost in the clinical setting and technical robustness and reproducibility outside expert centers. These 5 parameters were: monocyte HLA-DR by flow cytometry, percentage of immature neutrophils by flow cytometry (CD10^−^CD16^low^) [26], IL-6 and IL-10 concentration by enzyme-linked immunosorbent assay (ELISA) and total lymphocytes count by hemocytometer. Technical details may be found in the main study protocol [19].

### Determination of cutoff points

Receiver operating characteristic (ROC) curves were constructed for each of the 5 parameters at each timepoint, with the relevant clinical endpoint defined as secondary infection at day 30. Parameters with poor predictive ability (area under the curve (AUC) < 0.6 at each timepoint) were removed from the model and excluded from further analysis. For remaining parameters, optimal cutoff points were derived using the top left index (minimal distance to top left corner) and each parameter was further binarized into “low risk” and “high risk” at each timepoint.

Finally, pairwise Cox association models were performed, “adjusting” parameters with each other, thus giving a model for each pair of parameters to identify complementary or redundant ones. Redundant parameters were excluded from the model and from further analysis, to ensure that each parameter independently brought information to the model (excluding the redundant parameter with the lowest individual HR). A score was thus constructed as the combined sum of the binarized remaining parameters (with 1 point for “high risk”, and 0 point for “low risk” markers).

### Score

The predictive power of the resulting combination (score) was evaluated at the prespecified timepoint of interest D5–7 in patients still in ICU at that timepoint (cohort of interest), with absolute risk of secondary infection presented in each category. Univariate and adjusted Cox proportional hazards models were performed to adjust for a priori identified clinical risk factors for secondary infection, i.e. the physiological severity of illness (SOFA score at timepoint of interest), and disruption of normal barriers by invasive mechanical ventilation at timepoint of interest. Unadjusted and adjusted hazard ratios were computed.

Data are presented as numbers and percentages (qualitative variables) and medians and 25th/75th percentiles (quantitative variables). Chi square or Fisher’s exact test were used for qualitative variables assessment. Quantitative variables were compared with Mann–Whitney *U* test. The level of significance was set at 5%. Statistical analyses were computed with R software v3.6.2.

## Results

Out of 1079 screened patients, 353 (33%) were included in the main REALISM study [17]. The overall study population consisted primarily of males (65.4%) with a median age of 60 [47–71] years and an admission SAPS 2 score of 29 [20–43]. Of these, 278 patients (78.8%), 261 (73.9%) and 191 (54.1%) were still alive and in the ICU at timepoint D1–2, D3–4 and D5–7, respectively.

The cohort of interest (patients alive and still in ICU at D5–7) consisted of 189 patients after excluding individuals with missing data. From this cohort of interest, 42 (22.0%) developed subsequent secondary infections at D30 (Additional file 1: Table S1). The detailed description of patients’ characteristics is presented in Table 1.

**Table 1:** Patient characteristics

Preadmission characteristics	Whole REALISM cohort *n* = 353	Cohort of interest (ICU at D5–7) *n* = 189
Age (year)	60 [47–71]	59 [45–72]
Female gender	122 (34.6%)	52 (27.5%)
BMI (kg/m2)	25.0 [22.3–28.4]	25.2 [22.6–28.5]
Admission reason		
Sepsis*	107 (30.3%)	72 (38.1%)
Trauma	137 (38.8%)	95 (50.3%)
Surgery	109 (30.8%)	22 (11.6%)
Clinical characteristics		
Admission SAPS2	29 [20–43]	35 [23–47]
SOFA at admission	5 [1–8]	6 [2–9]
SOFA at D5–7	1 [0–2]	1 [0–4]
Mechanical ventilation at D5–7	33 (9.3%)	33 (18.2%)
Renal Replacement Therapy at D5–7	17 (4.8%)	16 (8.5%)
Outcomes		
Secondary infection at D 30	74 (21.0%)	42 (22.2%)
Secondary infection onset (days)	9 [6–15]	11 [9–17]
D30 ICU free days	23 [18–26]	22 [15.5–24]
D30 hosp. free days	15 [2–21]	9 [0–17.5]
D30 mortality	18 (5.1%)	10 (5.3%)

Data are presented as absolute numbers (percentage) or as median [Q1–Q3].

*See Additional file 1: Table S2 for detailed infection characteristics for patients admitted with sepsis.

### Predictive power of individual parameters

ROC curves were computed for each prespecified immune parameter (mHLA-DR, percentage of immature neutrophils, IL-6 and IL-10 concentration and total lymphocytes count, see e-Table 1). All parameters had an AUC above 0.6 at least at one timepoint except for lymphocyte count (AUC *0.46, 0.56 and 0.52,* respectively), which was thus excluded from further analysis. Cutoffs values were computed for the four remaining parameters (mHLA-DR, percentage of immature neutrophils, IL-6 and IL-10). For consistency, we only used the cutoff values computed at D5–7 from the cohort of interest. Values were dichotomized (“high-risk” vs “low-risk”). When adjusted for each other through pairwise association, every parameter brought complementary information to the models except for IL-6 and IL-10, which were considered redundant. Because IL-6 had the lowest predictive power for occurrence of secondary infection (unadjusted HR 1.82 (0.95–3.45), *p* = 0.069), it was excluded from further analysis.

The three remaining parameters were thus mHLA-DR, percentage of immature neutrophils and IL-10 (Table 2). When measured at days 5–7 from the cohort of interest, all three parameters had excellent predictive power for occurrence of secondary infection at day 30, with percentage of immature neutrophils performing best (unadjusted HR 2.55 (1.38–4.73) *p* = 0.003), followed by mHLA-DR (unadjusted HR 2.52 (1.31–4.85) *p* = 0.006), and IL-10 levels (HR 2.18 (1.07–4.45) *p* = 0.031).

**Table 2:** Parameters of the *REALIST* score and their predictive power of secondary infection at day 30

	Cutoff	AUC	Specificity (95% CI)	Sensitivity (95% CI)	PPV (95% CI)	NPV (95% CI)	HR (95% CI)
mHLA-DR	≤ 7627 Ab/C	0.71	0.63 (0.46–0.79)	0.73 (0.53–0.9)	0.27 (0.22–0.38)	0.92 (0.89–0.96)	2.52 (1.31–4.85)
Immature neutrophils	≥ 23.5%	0.71	0.72 (0.57–0.81)	0.7 (0.53–0.83)	0.32 (0.24–0.41)	0.93 (0.89–0.96)	2.55 (1.38–4.73)
IL-10	≥ 8.5 pg/ml	0.60	0.53 (0.43–0.69)	0.73 (0.53–0.87)	0.23 (0.19–0.29)	0.91 (0.87–0.96)	2.18 (1.07–4.45)

As measured in the cohort of interest (*n* = 189 ICU patients at days 5–7)

Values are presented with 95% confidence interval (CI)

*AUC* area under the curve, *PPV* positive predictive value, *NPV* negative predictive value

Predictive power for these three parameters was also computed for other prior timepoints (D1–2 and D3–4, see Additional file 1: Table S3). None of the individual parameters had significant predictive power for occurrence of secondary infection at D1–2. When measured at D3–4, parameters only had moderate predictive power for occurrence of secondary infection at day 30 (mHLA-DR, unadjusted HR 1.85, CI [1.15–2.98], *p* = 0.01; percentage of immature neutrophils, unadjusted HR 1.83 [1.12–3.00] *p* = 0.01); IL-10 levels, unadjusted HR 1.72, CI [1.07–2.77], *p* = 0.02).

### The REALIST score

**Fig. 1:**
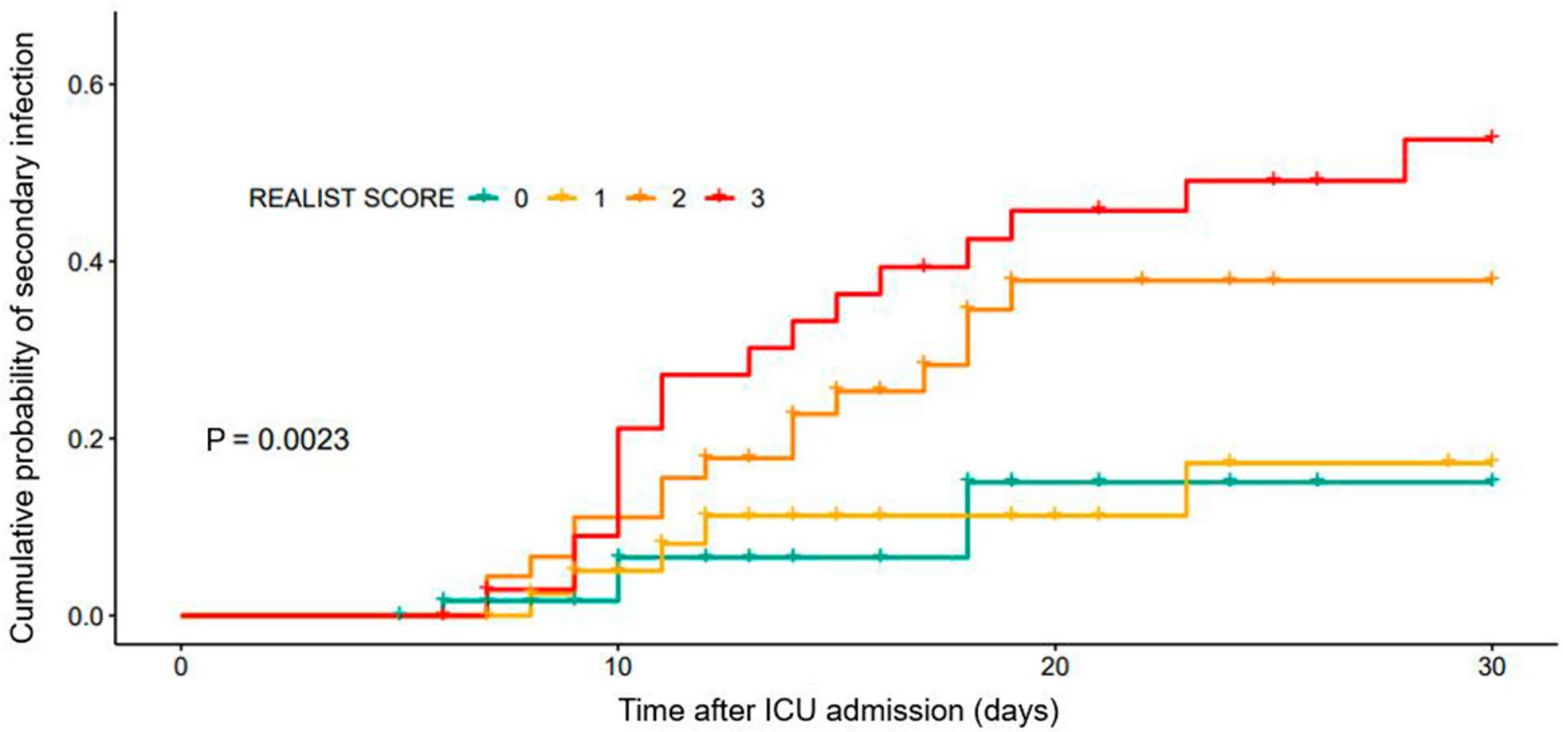
*REALIST* score and subsequent cumulative probability of secondary infection in survivors. Computed from Cox proportional hazards model in the cohort of interest (*n* = 189 ICU patients at days 5–7)

For these remaining parameters (mHLA-DR, immature neutrophils and IL-10), a point was given for each “high-risk” results as measured at D5–7. As such, for each patient still in the ICU at D5–7 (*n* = 189), a score between 0 and 3 was obtained, with 3 representing the highest risk of secondary infection. Incidence of secondary infection increased from 8% for patients with score of 0 to 46% in patients with a score of 3 (Fig. 1). Higher REALIST score was also associated with increased mortality at 30 days (*p* = 0.001 by Fisher's Exact Test).

When adjusted for a priori identified clinical risk factors for secondary infection, (SOFA score and invasive mechanical ventilation at timepoint of interest) a higher *REALIST* score was independently associated with increased risk of secondary infection (Table 3). For instance, patients with a score of 3 were 3.2 times more likely to develop secondary infection than patients with a score of 0, independent of clinical risk factors (adjusted HR 3.22 (1.09–9.50), *p* = 0.034).

**Table 3:** *REALIST* score and incidence of secondary infection and mortality at day 30

REALIST score	Secondary infection at D30					Mortality at D30
	Incidence	HR (95% CI)	*p*	Adjusted HR (95% CI)	p	
0 (*n* = 61)	5 (8.2%)	–	–	–	–	0 (0%)
1 (*n* = 42)	5 (11.9%)	1.15 (0.33–3.97)	0.827	1.05 (0.30–3.64)	0.943	0 (0%)
2 (*n* = 49)	15 (30.6%)	2.91 (1.05–8.03)	0.039	2.33 (0.81–6.73)	0.117	5 (10.2%)
3 (*n* = 37)	17 (46.0%)	4.41 (1.62–11.98)	0.003	3.22 (1.09–9.50)	0.034	5 (13.5%)

As measured in the cohort of interest (*n* = 189 ICU patients at days 5–7)

Hazard Ratios and *P* values were computed using univariate and adjusted Cox proportional hazards models from a priori identified clinical risk factors for secondary infection (SOFA score and mechanical ventilation at timepoint of interest)

Values are presented with 95% CI

## Discussion

As a sub-study of the REALISM project, the *REALIST* score was developed as a pragmatic and clinically applicable stratification strategy to identify patients with occult immune dysfunction. In our cohort of mixed ICU patients, the *REALIST* score was able to identify patients at high risk of secondary infections, an association that was independent from major clinical risk factors for infection. As such, this approach demonstrated that genuinely occult immune dysfunction can be identified in ICU patients with tools that are quite potentially available to the frontline critical care physician outside expert research centers [27, 28].

### Insights from the REALISM study

The REALISM study outlined how the immune response to injury engages all components of the immune system and does not significantly vary with the type of injury (infectious vs sterile). The initial response is not associated with increased risk of death and secondary infection, illustrating that the initial pro-inflammatory immune response induced by injury should not necessarily be seen as a deleterious factor per se but rather represents an adaptive response to the injury. As induction of the pro-inflammatory effector response is associated with the concomitant development of regulatory mechanisms to protect the host from such overwhelming immune response, this also illustrates the complex interplay between the effector and regulatory mechanisms of the immune system to set up a coordinated immune response to injury. This initial host response likely aims at controlling the aggression and at protecting the host from deleterious off-targets effects of this tremendous immune response.

Thus, after the initial physiologic immune response to injury, it is the persistence (or delayed recovery) of immune alterations that predisposes patients to deleterious infectious events, independently of usual confounding factors. In this subgroup of patients, this persistently dysregulated immune profile cannot be considered as part of the physiologic response to injury but rather as a maladaptive evolution of the immune response.

Therefore, as tempting it might be to try to predict subsequent infection in patients soon after ICU admission, this might not be neither practical nor pertinent. A promising approach to immune monitoring, therefore, seems to be to target the persistence of immune alterations at the end of the first week of ICU stay, identifying patients in which immune homeostasis is pathologically compromised. Knowledge of such occult immune dysfunction is not only interesting, it can also directly influence and hopefully optimize patients’ care, either through enhanced clinical surveillance, accelerated start of antimicrobials in case of infection suspicion, removal of potentially superfluous invasive device, and eventually through immune stimulation strategies [29]. In parallel, a *REALIST* score of 0 in an otherwise clinically stable patient would provide further reassurance and possibly support deescalating antimicrobial treatment in the right clinical context, for instance. Finally, such immune function scoring could be used to enrich a study population with patients at high risk for secondary infection in the context of an eventual immune stimulation randomized controlled trial.

Our study echoes the work of Conway-Moris et al.in the important INFECT study [14], in which the authors elegantly presented an immune score based on levels of mHLA-DR, Treg lymphocytes (CD4^+^/CD25^++^/CD127^−^) and dysfunctional neutrophils (nCD88) by flow cytometry after strict standardization within different centers. Their score was shown to predict secondary infection in critically ill patients with organ dysfunction (unadjusted HR 4.30 [CI 1.70–10.20] when measured at days 4–6), which interestingly is quite similar to the performance of the *REALIST* score (unadjusted HR 4.41 [CI 1.63–11.98] at D5–7). Even though Conway-Morris et al. selected different immune parameters than ours and even though patients in the INFECT study had higher illness severity than patients in REALISM, these similar results tend to validate the concept and pertinence of combining parameters to tackle immune monitoring in the ICU across a wide range of patients, even those whom clinical status might seem somewhat reassuring.

Recently, Fang et al. described and validated a similar immune dysfunction score performed at day 1 of ICU admission to help predict mortality in critically ill patients. In that study [30], the combination of monocyte HLA-DR, Il-10 levels, G-CSF levels and ratio of segmented neutrophils to monocyte allowed predicted 28 day mortality with an AUC of 0.789. Interestingly, even though the timepoint and outcome of interest are different than those in the *REALIST* score, there is significant similarities between chosen immune parameters, further reinforcing the rationale behind immune monitoring in the critically ill.

### Choice of immune parameters

Besides mHLA-DR, which is the most studied and validated biomarker in the field with widespread standardization across laboratories [31, 32], Conway-Morris et al. also used the level of Treg (CD4^+^/CD25^++^/CD127^−^) and neutrophils surface expression of CD88. Neutrophil CD88, a receptor for complement anaphylatoxin C5a, has been relatively scarcely described in the critical care context and has only been reported in expert research centers [33–35]; it has not been performed in the REALISM study. In parallel, the phenotypical identification of Treg lymphocytes is notoriously problematic and the standardization of their staining by flow cytometry is challenging even with modern techniques in expert centers [36–38]. Of note, in the REALISM study, percentage of Treg lymphocytes was not associated with occurrence of secondary infection at D30 through univariate analysis, whether measured at days 1–2, 3–4 or 5–7 (best HR at D3–4: 1.08 (0.84–1.38), *p* = 0.552).

As immunophenotyping has historically suffered from lack of standardization and reproducibility [39], particular importance must be attributed to these aspects if one hopes for immune monitoring tools to permeate into clinical practice. Thus, for the *REALIST* score, we purposely and pre-emptively chose immune parameters based on their immediate applicability in clinical practice outside expert centers. As such, technical robustness and reproducibility were major drivers for selecting otherwise relevant immune parameters from the REALISM study.

To complement mHLA-DR, we selected the expression of CD10 and CD16 on neutrophils as a technically simple marker of dysregulated granulopoiesis and inadequate granulocyte maturation. In other words, CD10^low^CD16^low^ neutrophils are immature and quite probably the immunophenotypic equivalent of band cells [26, 40, 41], although variability and impreciseness in band cell measurement [42] has precluded such a definite association. Like band cells [43], an increase in CD10^low^CD16^low^ neutrophils has been associated with poor outcomes in sepsis patients, namely, occurrence of secondary infection and death [41], and might also directly contribute to impaired T cell function [40]. Our study supports these past findings, as higher proportion of circulating CD10^low^CD16^low^ neutrophils at days 5–7 was independently associated with occurrence of secondary infection at day 30.

Lymphopenia has been found to be associated with poor outcome after sepsis and in other clinical illnesses, and therapeutic interventions to increase lymphocytes levels after sepsis have been proposed and are under investigation [44]. Surprisingly, low lymphocytes levels were not associated with secondary infection or mortality at any timepoint in the REALISM cohort, a finding that might be due to lower severity of illness and relatively low event rate.

IL-6 and IL-10 levels were both associated with occurrence of secondary infection in our study, although they brought redundant information in pairwise analysis. As IL-6 is a pro-inflammatory cytokine and IL-10 a globally anti-inflammatory cytokine, it was somewhat expected that elevated IL-10 levels would be associated with immune dysfunction, which was confirmed in this study. Of note, IL-10 levels are strongly correlated with IL-6 levels at all timepoints, as shown in the REALISM study [17], a finding that reflects the intricate and immediate interplay between effector and regulatory limbs of the immune system. As such, IL-6 levels that fail to return towards normal at days 5–7 suggest either immune dysfunction through impaired homeostasis mechanisms and/or uncontrolled inflammatory focus with associated higher disease severity. Of note, the latter hypothesis is supported by the finding that the association between higher IL-6 levels at days 5–7 and secondary infection was not statistically significant after controlling for SOFA score and presence of invasive device.

### Strengths and limitations

In our study, we assessed the performance of multiple parameters at multiple timepoints in a relatively large cohort of mixed critically ill patients. We tailored our score to be easily applicable in clinical practice, with a fixed and clear timepoint, reliable and technically robust parameters with a strong track record, and simple computation by bedside clinicians. We also demonstrated strong association with secondary infections even after controlling for SOFA score and presence of invasive mechanical ventilation. We elected to control for both of these variables, even though they are partially redundant (as respiratory support is included in the SOFA score) because of the strong and clinically important association between invasive mechanical ventilation and risk of infection (namely, pneumonia). This thus represents a more stringent statistical correction than seen in other similar studies, further supporting the claim that our score is not a mere marker of disease severity but really an immune monitoring tool that provides genuinely new and previously occult information to the clinician.

Among significant limitations, our study was single-centre and suffers from a relatively low disease severity and low event rate. Although this reduced the strength of association between parameters and outcome, it also suggests that the score is applicable to a wide array of patients with varying disease severity, as secondary infections can occur even in patients with low disease severity with genuinely occult immune dysfunction. Importantly, the *REALIST* score will have to be further validated in a separate multicentric cohort.

## Interpretation

In conclusion, we derived and presented the *REALIST* score, a simple and pragmatic stratification strategy which provides critical care clinicians with a clear and useful assessment of the occult immune status of their patient. This new tool could help optimize care of these fragile individuals and could contribute in designing future trials of immune stimulation strategies. Ultimately, we believe this score, in conjunction with the main REALISM study, provides important didactic value, as the question of critical illness induced immune dysfunction warrants widespread discussion within the critical care community if we are to adapt our practice to this complex phenomenon and lastingly provide better care.

## Supplementary Information

**Additional file 1: Table S1.** Secondary infection characteristics (n=42) in the cohort of interest. **Table S2.** Description of sites and types of initial infections in the subgroup of septic patients. **Table S3.** Individual predictive power (for occurrence of secondary infection) of immune parameters at different timepoints for patients still in the ICU at day 5–7 (n=189). **Table S4.** Sample of different cut-off points for mHLA-DR ROC-curve and their respective predictive power for secondary infection at D30. **Table S5.** Sample of different cut-off points for percentage of immature neutrophils ROC-curve and their respective predictive power for secondary infection at D30.

## Data Availability

Original data will be made available upon adequate request to the corresponding author.

## Abbreviations

AUC: Area under the curve
BMI: Body mass index
CI: Confidence interval
EDTA: Ethylenediaminetetraacetic acid
ELISA: Enzyme-linked immunosorbent assay
ICU: Intensive care unit
mHLA-DR: Monocyte human leucocyte antigen-DR\
NPV: Negative predictive value
PPV: Positive predictive value
ROC: Receiver operating characteristic
SAPS: Simplified Acute Physiological Score
SOFA: Sequential Organ Failure Assessment
