# Supplementary material for: A stratification strategy to predict secondary infection in critical illness-induced immune dysfunction: the REALIST score
Source: Ann Intensive Care. 2022 Aug 17;12:76. doi: 10.1186/s13613-022-01051-3 (PMC9382015; doi:10.1186/s13613-022-01051-3)
Supplement: Supplementary file 1 — Additional file 1: Table S1. Secondary infection characteristics (n=42) in the cohort of interest. Table S2. Description of sites and types of initial infections in the subgroup of septic patients. Table S3. Individual predictive power (for occurrence of secondary infection) of immune parameters at different timepoints for patients still in the ICU at day 5–7 (n=189). Table S4. Sample of different cut-off points for mHLA-DR ROC-curve and their respective predictive power for secondary infection at D30. Table S5. Sample of different cut-off points for percentage of immature neutrophils ROC-curve and their respective predictive power for secondary infection at D30. [file 13613_2022_1051_MOESM1_ESM.docx]

**A stratification strategy to predict secondary infection in critical illness-induced immune dysfunction: the REALIST score**

Short Title: The REALIST score

Jan-Alexis Tremblay MD^1,2^, Florian Perron MSc^1^, Louis Kreitmann MD^1^, Julien Textoris MD PhD^1^, Karen Brengel-Pesce PhD^1^, Anne-Claire Lukaszewicz MD PhD^1,3^, Laurence Quemeneur PhD^4^, Christophe Vedrine PhD^5^, Lionel K Tan FRCP^6^, Fabienne Venet PhD^7,8^, Thomas Rimmelé MD PhD^1,3^, Guillaume Monneret PhD^1,7^ for the REALISM study group

**Supplementary Material**


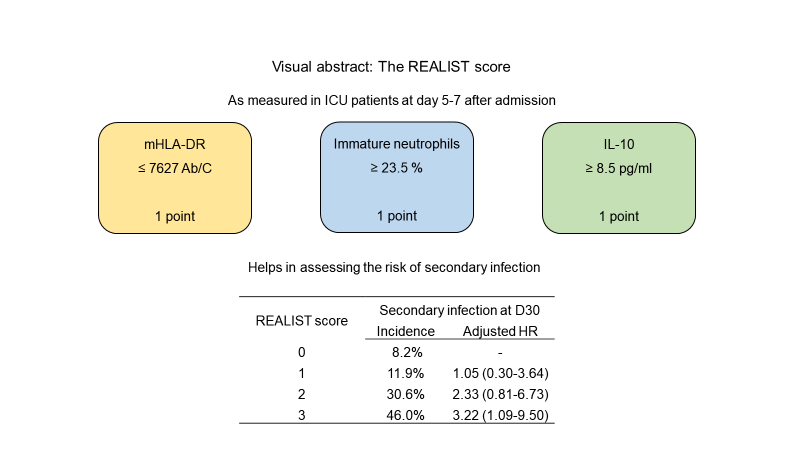


Table S1. Secondary infection characteristics (n=42) in the cohort of interest

| Probability |  |
| --- | --- |
| Likely | 22 (52%) |
| Definite | 20 (48%) |
| Site of infection |  |
| Pneumonia | 12 (29%) |
| Intra abdominal | 6 (14%) |
| Urinary tract | 8 (19%) |
| Blood stream / catheter | 8 (19%) |
| Surgical site | 4 (10%) |
| Other | 4 (10%) |
| Identified microorganism |  |
| Yes | 36 (86%) |
| No | 6 (14%) |

As measured in the cohort of interest (n=189 ICU patients at day 5-7). Infections were noted up to day 30 after ICU admission.

Table S2 Description of sites and types of initial infections in the subgroup of septic patients:

| **Variable** | **Sepsis  n=72** |
| --- | --- |
| Intra-abdominal infection | 29 (40 %) |
| Pneumonia | 20 (28 %) |
| Skin and soft tissue | 6 (8.3 %) |
| Urinary infection | 5 (6.9 %) |
| Bone and articulation | 3 (4.2 %) |
| Surgical site infection | 2 (2.8 %) |
| Catheter-related | 1 (1.4 %) |
| Other | 6 (8.3 %) |
| Infection type  *Community acquired*  *Hospital acquired* | 51 (71 %)  21 (29 %) |
| Positive bacteremia | 28 (39 %) |
| Identified micro-organism |  |
| *Enterobacteriaceae* | 21 (29 %) |
| *Pseudomonas/Stenotrophomonas* | 1 (1.4 %) |
| *Acinetobacter* | 1 (1.4 %) |
| *Other Non Enterobacteriaceae Gram - Bacilli* | 1 (1.4 %) |
| *Streptococcus* | 2 (2.8 %) |
| *Enterococcus* | 8 (11.1 %) |
| *Staphylococcus* | 9 (12.5 %) |
| *Gram+ Bacilli* | 1 (1.4 %) |
| *Candida* | 7 (9.7 %) |
| *Aspergillus* | 3 (4.2 %) |

Table S3. Individual predictive power (for occurrence of secondary infection) of immune parameters at different timepoints for patients still in the ICU at day 5-7 (n=189)

| **Marker** | **Time point** | **AUC** | **Cut-off (95% CI)** | **Specificity (95% CI)** | **Sensitivity (95% CI)** | **PPV (95% CI)** | **NPV (95% CI)** |
| --- | --- | --- | --- | --- | --- | --- | --- |
| mHLA-DR | D1-D2 | 0,51 | 5615 (4041,9-7723,2) | 0,54 (0,35-0,72) | 0,59 (0,37-0,81) | 0,19 (0,14-0,26) | 0,88 (0,83-0,93) |
| mHLA-DR | D3-D4 | 0,65 | 5928,7 (4286,8-6145,3) | 0,63 (0,53-0,8) | 0,67 (0,5-0,83) | 0,27 (0,21-0,38) | 0,91 (0,86-0,95) |
| mHLA-DR | D5-D7 | 0,71 | 7626,5 (5546,3-9611,4) | 0,63 (0,46-0,79) | 0,73 (0,53-0,9) | 0,27 (0,22-0,38) | 0,92 (0,89-0,96) |
| Immature neutrophils | D1-D2 | 0,61 | 59,6 (41,5-72,7) | 0,58 (0,44-0,72) | 0,7 (0,52-0,85) | 0,23 (0,18-0,3) | 0,91 (0,87-0,95) |
| Immature neutrophils | D3-D4 | 0,65 | 21,3 (19,6-42,6) | 0,56 (0,46-0,76) | 0,73 (0,5-0,87) | 0,26 (0,2-0,35) | 0,91 (0,87-0,96) |
| Immature neutrophils | D5-D7 | 0,71 | 23,5 (13,5-29,5) | 0,72 (0,57-0,81) | 0,7 (0,53-0,83) | 0,32 (0,24-0,41) | 0,93 (0,89-0,96) |
| IL-6 | D1-D2 | 0,62 | 269,8 (122,8-389) | 0,69 (0,48-0,78) | 0,63 (0,44-0,81) | 0,26 (0,19-0,35) | 0,91 (0,87-0,95) |
| IL-6 | D3-D4 | 0,62 | 68,9 (65,7-85,8) | 0,59 (0,51-0,69) | 0,73 (0,6-0,87) | 0,27 (0,22-0,33) | 0,92 (0,88-0,96) |
| IL-6 | D5-D7 | 0,61 | 42 (30,8-47,2) | 0,57 (0,46-0,67) | 0,7 (0,57-0,87) | 0,24 (0,19-0,3) | 0,91 (0,87-0,95) |
| IL-10 | D1-D2 | 0,6 | 17,4 (15,3-42,2) | 0,55 (0,44-0,81) | 0,67 (0,48-0,85) | 0,22 (0,17-0,32) | 0,91 (0,86-0,95) |
| IL-10 | D3-D4 | 0,61 | 12,4 (11,1-14,1) | 0,59 (0,49-0,68) | 0,73 (0,6-0,87) | 0,27 (0,22-0,33) | 0,92 (0,88-0,96) |
| IL-10 | D5-D7 | 0,6 | 8,5 (7,5-11,2) | 0,53 (0,43-0,69) | 0,73 (0,53-0,87) | 0,23 (0,19-0,29) | 0,91 (0,87-0,96) |
| Lymphocyte count | D1-D2 | 0,43 | 1018 (382-1461,5) | 0,48 (0,24-0,97) | 0,48 (0,19-0,7) | 0,16 (0,11-0,62) | 0,85 (0,78-0,9) |
| Lymphocyte count | D3-D4 | 0,55 | 1023 (748-1146,5) | 0,5 (0,37-0,75) | 0,67 (0,4-0,83) | 0,22 (0,17-0,31) | 0,88 (0,84-0,93) |
| Lymphocyte count | D5-D7 | 0,51 | 982 (803,5-1151) | 0,58 (0,39-0,79) | 0,53 (0,33-0,73) | 0,2 (0,14-0,29) | 0,87 (0,82-0,91) |

Table S4

Sample of different cut-off points for mHLA-DR ROC-curve and their respective predictive power for secondary infection at D30.

| Cut-off | Sensitivity | Specificity | PPV | NPV |
| --- | --- | --- | --- | --- |
| 70073 | 1.00 | 0.01 | 0.16 | 1.00 |
| 20669 | 1.00 | 0.07 | 0.17 | 1.00 |
| 17147 | 1.00 | 0.14 | 0.18 | 1.00 |
| 15430 | 1.00 | 0.21 | 0.20 | 1.00 |
| 13904 | 0.97 | 0.26 | 0.20 | 0.98 |
| 11919 | 0.90 | 0.32 | 0.20 | 0.94 |
| 10464 | 0.90 | 0.39 | 0.22 | 0.95 |
| 9611 | 0.90 | 0.45 | 0.24 | 0.96 |
| 8792 | 0.80 | 0.50 | 0.24 | 0.93 |
| 8014 | 0.73 | 0.55 | 0.24 | 0.91 |
| **7627** | **0.67** | **0.60** | **0.24** | **0.90** |
| 7098 | 0.60 | 0.65 | 0.25 | 0.89 |
| 6229 | 0.53 | 0.72 | 0.27 | 0.89 |
| 5610 | 0.47 | 0.77 | 0.29 | 0.88 |
| 4928 | 0.33 | 0.82 | 0.26 | 0.86 |
| 4250 | 0.33 | 0.89 | 0.37 | 0.87 |
| 3441 | 0.17 | 0.93 | 0.31 | 0.85 |
| 882 | 0.00 | 0.99 | 0.00 | 0.84 |

The cut-off at 7627 was selected based on the top-left index to optimize both sensitivity and specificity

Table S5

Sample of different cut-off points for percentage of immature neutrophils ROC-curve and their respective predictive power for secondary infection at D30.

| Cut-off | Sensitivity | Specificity | PPV | NPV |
| --- | --- | --- | --- | --- |
| 0.42 | 1.00 | 0.01 | 0.16 | 1.00 |
| 2.27 | 1.00 | 0.08 | 0.17 | 1.00 |
| 3.24 | 0.93 | 0.14 | 0.17 | 0.91 |
| 4.13 | 0.90 | 0.19 | 0.18 | 0.91 |
| 5.20 | 0.90 | 0.26 | 0.19 | 0.93 |
| 6.05 | 0.87 | 0.32 | 0.20 | 0.93 |
| 6.77 | 0.87 | 0.39 | 0.21 | 0.94 |
| 8.12 | 0.80 | 0.45 | 0.22 | 0.92 |
| 10.99 | 0.77 | 0.50 | 0.23 | 0.92 |
| 12.99 | 0.77 | 0.57 | 0.26 | 0.93 |
| 15.56 | 0.67 | 0.61 | 0.25 | 0.90 |
| 19.31 | 0.67 | 0.67 | 0.28 | 0.91 |
| **23.50** | **0.67** | **0.72** | **0.32** | **0.92** |
| 26.85 | 0.53 | 0.76 | 0.30 | 0.89 |
| 32.36 | 0.47 | 0.82 | 0.33 | 0.89 |
| 36.54 | 0.40 | 0.87 | 0.38 | 0.88 |
| 48.05 | 0.23 | 0.91 | 0.33 | 0.86 |
| 67.73 | 0.07 | 0.95 | 0.20 | 0.84 |
| 98.52 | 0.03 | 1.00 | 1.00 | 0.84 |

The cut-off at 23.50 was selected based on the top-left index to optimize both sensitivity and specificity

Table S6

Sample of different cut-off points for IL-10 ROC-curve and their respective predictive power for secondary infection at D30.

| Cut-off | Sensitivity | Specificity | PPV | NPV |
| --- | --- | --- | --- | --- |
| 2.83 | 1.00 | 0.01 | 0.16 | 1.00 |
| 4.22 | 0.97 | 0.06 | 0.17 | 0.91 |
| 4.76 | 0.97 | 0.13 | 0.18 | 0.95 |
| 5.35 | 0.97 | 0.19 | 0.19 | 0.97 |
| 5.66 | 0.90 | 0.25 | 0.19 | 0.93 |
| 6.21 | 0.83 | 0.30 | 0.19 | 0.90 |
| 6.61 | 0.83 | 0.36 | 0.20 | 0.92 |
| 7.20 | 0.83 | 0.43 | 0.22 | 0.93 |
| 7.61 | 0.80 | 0.47 | 0.23 | 0.92 |
| **8.50** | **0.73** | **0.52** | **0.23** | **0.91** |
| 9.53 | 0.60 | 0.55 | 0.21 | 0.88 |
| 10.32 | 0.53 | 0.61 | 0.21 | 0.87 |
| 11.54 | 0.43 | 0.66 | 0.20 | 0.86 |
| 12.35 | 0.33 | 0.71 | 0.18 | 0.85 |
| 13.68 | 0.23 | 0.75 | 0.16 | 0.84 |
| 16.20 | 0.17 | 0.81 | 0.14 | 0.83 |
| 20.31 | 0.13 | 0.86 | 0.16 | 0.84 |
| 26.05 | 0.10 | 0.92 | 0.20 | 0.84 |
| 42.23 | 0.07 | 0.98 | 0.40 | 0.84 |
| 134.01 | 0.00 | 0.99 | 0.00 | 0.84 |

The cut-off at 8.50 was selected based on the top-left index to optimize both sensitivity and specificity
